# Clinical Characteristics and Prognosis of Hemolytic Disease of the Newborn Caused by Irregular Antibodies: A 13-Year Retrospective Analysis

**DOI:** 10.3390/children11121409

**Published:** 2024-11-21

**Authors:** Hui Wu, Rui Li, Hongling Wei, Weiwei Zhu, Yan Xing

**Affiliations:** Department of Pediatrics, Peking University Third Hospital, Beijing 100191, China; xiaoyiwh107@126.com (H.W.); lirui8588@126.com (R.L.); 13810262688@126.com (H.W.); yeshangwuzhebang@163.com (W.Z.)

**Keywords:** hemolytic disease of the newborn, neonatal jaundice, anemia, prognosis, screening, neonatal outcomes

## Abstract

Background/Objectives: The clinical characteristics and outcomes of hemolytic disease of the newborn (HDN) caused by irregular antibodies remain unclear. Herein, we analyzed the clinical features and prognosis of HDN. Methods: Children admitted to our institution between June 2009 and December 2022 with a definite diagnosis of HDN were evaluated. Patients with irregular antibodies were matched in a 1:3 ratio to those with ABO incompatibility. Children with confirmed Rh-incompatibility hemolytic disease were divided into the RhD subgroup (hemolysis induced by Rh anti-D) and the non-RhD group (hemolysis induced by other Rh antibodies). Results: The irregular antibody and ABO incompatibility group included 32 and 96 patients, respectively. Compared to the ABO incompatibility group, the irregular antibody group showed earlier jaundice; higher incidence of liver and spleen enlargement and anemia; higher direct antiglobulin test (DAT) positivity; earlier and more severe anemia; higher rates of enhanced phototherapy, blood transfusion, and blood exchange; and longer hospital stay (all *p* < 0.05). Compared to the non-RhD group, the RhD subgroup showed an earlier occurrence of jaundice and a higher incidence of liver and spleen enlargement (both *p* < 0.05). The multiple irregular antibody subgroup further showed earlier occurrence of jaundice and a higher rate of enhanced phototherapy, blood transfusion, and blood exchange than the single-antibody group (both *p* < 0.05). Conclusions: HDN caused by irregular red blood cell antibodies is rare, but clinical manifestations are serious. It is important to pay attention to the screening of irregular antibodies during pregnancy, to strengthen monitoring, and to provide intrauterine treatment and early intervention as necessary.

## 1. Introduction

Hemolytic disease of the newborn (HDN) refers to an immune hemolytic disease caused by blood group incompatibility between a mother and fetus. It is a common cause of neonatal jaundice and anemia. Thus far, over 40 blood group systems and 350 red blood cell group antigens have been identified, all of which have immunogenic potential [1]. The most prevalent type of HDN is ABO incompatibility, followed by Rh incompatibility. HDN can also occur in other less common red blood group systems, such as Kell, Kidd, and MNS [2]. Diagnosis of irregular antibodies outside of the ABO system requires specialized laboratory tests, making cases prone to being overlooked or misdiagnosed. This can result in unnecessary testing, adverse outcomes, and increased economic burden [3]. Following advancements in diagnostic techniques, more cases of HDN due to irregular antibodies are being diagnosed and recognized in clinical practice. HDN not only causes neonatal jaundice and anemia but may also result in worse conditions, such as prolonged or aplastic anemia [3]. This increases the psychological and social burden for parents and negatively affects early childhood health. Nevertheless, there is currently limited research on the maternal factors, clinical characteristics, and outcomes of HDN caused by irregular antibodies. Therefore, the present study aimed to analyze the maternal conditions, clinical characteristics, and prognosis of patients with HDN caused by irregular antibodies, with patients with ABO incompatibility HDN as reference.

## 2. Materials and Methods

### 2.1. Study Design and Participants

This retrospective study evaluated newborns diagnosed with HDN admitted to the neonatal intensive care unit or neonatal ward at Peking University Third Hospital between 1 June 2009 and 30 November 2022. Patients with incomplete clinical data due to voluntary discharge and those with other potential causes of hemolysis (e.g., genetic metabolic disorders; severe infections; or hemolysis caused by RBC enzyme, membrane, or hemoglobin abnormalities) were excluded.

### 2.2. Variable Definition

HDN was defined as an alloimmune hemolytic disease of the newborn caused by maternal–fetal blood group incompatibility [2]. The diagnostic criteria for neonatal hyperbilirubinemia and acute bilirubin encephalopathy were based on the Expert Consensus on the Diagnosis and Treatment of Neonatal Hyperbilirubinemia [4]. Hearing impairment was characterized by prolonged latencies in brainstem auditory evoked potentials or hearing loss. Hyperbilirubinemia-related imaging abnormalities refer to abnormal signals or echoes in the basal ganglia or globus pallidus detected by cranial magnetic resonance imaging or ultrasound during the acute phase without clinical signs of acute bilirubin encephalopathy. Late-onset anemia was defined as anemia appearing or persisting between 1 week and 3 months after birth [3].

### 2.3. Patient Grouping

Patients were divided into two groups based on the type of red blood cell (RBC) antibodies: the ABO incompatibility group and the irregular antibody group. The ABO incompatibility group involved newborns diagnosed with ABO incompatibility hemolytic disease, whereas the irregular antibody group involved newborns diagnosed with Rh or other rare blood group incompatibility hemolytic disease. The Rh incompatibility group was further divided into two subgroups based on the type of RBC antibody: the RhD subgroup (hemolysis caused by Rh anti-D antibodies) and the non-RhD subgroup (hemolysis caused by other Rh antibodies, such as anti-c, anti-C, anti-E, or anti-e antibodies). The irregular antibody group was further divided into two subgroups based on the number of antibody types involved, namely, the single irregular antibody subgroup involving patients with hemolysis caused by a single irregular antibody and the multiple irregular antibodies group involving patients with hemolysis caused by two or more irregular antibodies. The irregular antibody group was propensity-matched in a 1:3 ratio with a control group of newborns with similar gestational age and birth weight and who were diagnosed with ABO incompatibility hemolytic disease during the same period.

### 2.4. Data Collection

The following clinical data were collected: General information included sex, gestational age, birth weight, maternal gravidity and parity, previous pregnancy history, and maternal complications during pregnancy. Clinical information included the onset of jaundice and the presence of hepatosplenomegaly, hyperbilirubinemia, and anemia. Treatment and outcome data included the type of treatment: standard phototherapy (light intensity 8–10 μW/[cm^2^·nm]), intensive phototherapy (light intensity 30 μW/[cm^2^·nm]), intravenous immunoglobulin (IVIG), intravenous albumin infusion, blood transfusion, and exchange transfusion. Outcomes included systemic complications, length of hospital stay, and the presence of bilirubin encephalopathy, hearing impairment, and hyperbilirubinemia-related imaging abnormalities. Laboratory data included parental and newborn blood types, peak total bilirubin (TBIL, Enzymatic method) levels and time to the occurrence, hemoglobin (HGB, automated hematology analyzer) nadir and time to occurrence, peak reticulocyte (RET, Semiconductor laser flow cytometry) and time to occurrence, peak coefficient of variation of RBC distribution width (RDW-CV, automated hematology analyzer) and time to occurrence, direct antiglobulin test (DAT, microtube gel technique) result, indirect antiglobulin test (IAT, microtube gel technique) result, maternal monitoring of irregular antibodies during pregnancy, and fetal middle cerebral artery peak systolic velocity (MCA-PSV). Prognostic indicators included changes in hemoglobin levels and treatment within the first 3 months after birth, assessed to determine the occurrence and severity of late-onset anemia.

### 2.5. Statistical Analysis

Normally distributed measurement data are expressed as the means ± standard deviations ($\bar{x}$ ± s), whereas non-normally distributed data are expressed as the medians (interquartile ranges). Categorical data are presented as frequencies (percentages). Normally distributed measurement data were compared between two groups using the t-test, whereas non-normally distributed data were compared using the rank-sum test. Pairwise comparisons of categorical data, expressed as number of patients (%), were conducted using the chi-squared test or Fisher’s exact test. Propensity score matching was employed for subject matching (utilizing a caliper of 0.2 times the standard deviation, with nearest-neighbor matching). All statistical analyses were performed using SPSS statistical software version 25.0 (Armonk, NY, USA: IBM Corp). A *p*-value of <0.05 was considered significant.

## 3. Results

### 3.1. Comparison Between the ABO Incompatibility and Irregular Antibody Groups

#### 3.1.1. Maternal Gestational Conditions and General Information

**Figure 1:**
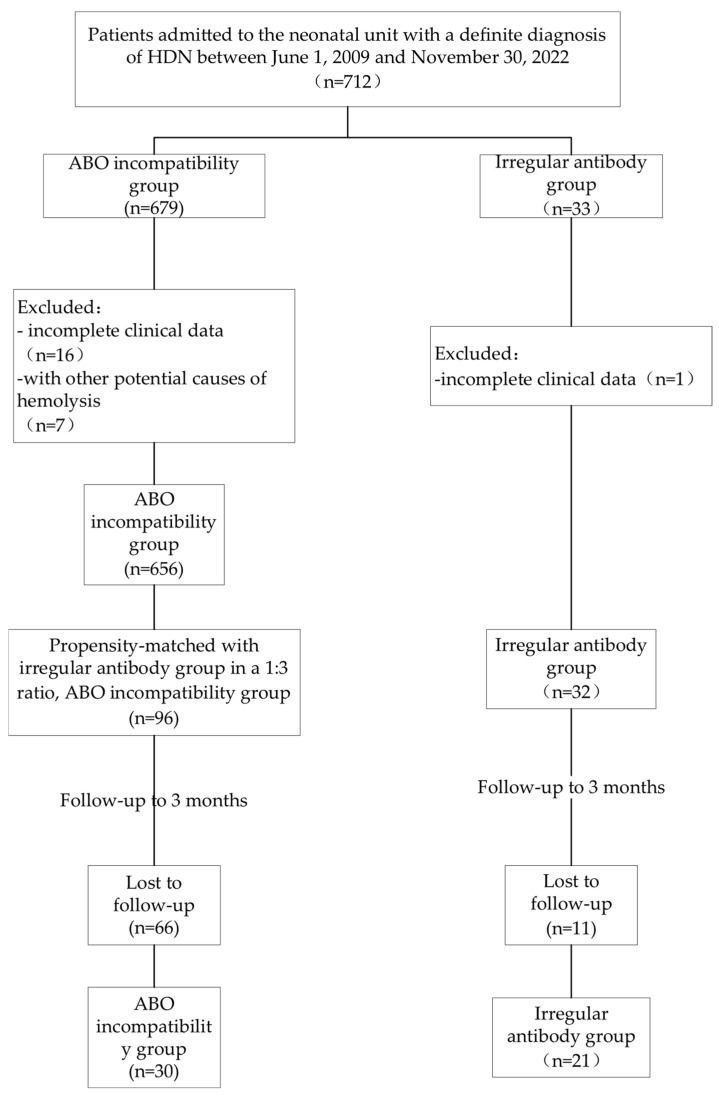
Flowchart of patient selection and follow-up.

A total of 688 patients were diagnosed with HDN during the 13-year study period. The irregular antibody group accounted for 4.6% (32/688) of the cohort and included 1 patient with MNS incompatibility hemolytic disease, 2 with Kidd incompatibility hemolytic disease, 20 with RhD, 8 with non-RhD, and 1 with Kidd combined with Rh incompatibility. Consequently, a total of 96 patients with ABO incompatibility hemolytic disease were propensity-matched in a 1:3 ratio. The patient inclusion, grouping, and follow-up are illustrated in Figure 1.

With respect to maternal gravidity and parity, compared with the ABO incompatibility group, the irregular antibody group included a significantly higher proportion of mothers with a history of previous pregnancies (first pregnancy: 3.23% vs. 34.38%; first delivery: 22.58% vs. 67.71%; both *p* < 0.001). In the irregular antibody group, 31 (96.87%) mothers exhibited positive results for irregular antibody screening during pregnancy, and 16 (51.61%) mothers underwent MCA-PSV monitoring, with 3 mothers exceeding the intervention threshold (>1.5 Multiples of the Median, MoM). All three of the mothers underwent preterm cesarean sections, and one mother received an intrauterine transfusion. In the RhD group, two mothers received anti-D antibody treatment. Regarding maternal pregnancy complications, both groups included a total of 32 cases of gestational diabetes, 22 cases of gestational hypertension-related diseases, 10 cases of anemia, 8 cases of thyroid disorders and premature rupture of membranes, and 3 cases of antiphospholipid antibody syndrome; the differences in the proportions of various diseases between the two groups were not statistically significant. Further, no statistically significant differences were observed in gestational age (38.25 weeks [36.9, 39.32 weeks] vs. 37.75 weeks [36.08, 39.5 weeks]) or birth weight (3120 g [2600, 3525 g] vs. 2980 g [2587.5, 3392.5 g]) between the ABO incompatibility and the irregular antibody groups (both *p* > 0.05).

#### 3.1.2. Clinical Manifestations

The irregular antibody group developed jaundice significantly earlier than the ABO incompatibility group and exhibited a significantly higher proportion of patients with hepatosplenomegaly and anemia (both *p* < 0.05, Table 1). However, the proportions of patients with neonatal hyperbilirubinemia and with bilirubin encephalopathy were not significantly different between the two groups (both *p* > 0.05).

#### 3.1.3. Laboratory Results

Compared with the ABO incompatibility group, the irregular antibody group demonstrated a significantly higher positive rate for the DAT, more severe anemia along with an earlier occurrence, and earlier peak times for RET and RDW-CV (all *p* < 0.05, Table 2). Both groups exhibited a 100% positivity rate for the IAT.

#### 3.1.4. Treatment Modalities and Outcomes

Compared with the ABO incompatibility group, the irregular antibody group had a significantly higher proportion of patients who received intensive phototherapy, blood transfusions, and exchange transfusions and significantly longer hospital stay (all *p* < 0.05, Table 3). Conversely, the proportions of patients who received IVIG or the incidence of hearing abnormalities and imaging anomalies showed no significant differences between the two groups (both *p* > 0.05).

### 3.2. Comparison Between the RhD and Non-RhD Groups in Rh Incompatibility Hemolytic Disease

#### 3.2.1. General Information

Among the 32 patients with irregular antibodies, 20 (62.5%) and 8 (25%) belonged to the RhD and non-RhD subgroups, respectively. Gestational age was not significantly different between the two groups (37 weeks [35.55, 38.67 weeks] vs. 39.1 weeks [38.58, 40.2 weeks], *p* > 0.05). The non-RhD subgroup involved four, one, one, and two patients with anti-E, anti-C + anti-E, anti-C + anti-E, and anti-E + anti-C-induced hemolysis, respectively. However, the RhD subgroup involved 17, 2, and 1 patients with anti-D, anti-C + anti-D, and anti-C + anti-D + anti-E-induced hemolysis, respectively.

#### 3.2.2. Clinical Manifestations

Compared with the non-RhD group, the RhD group experienced significantly earlier onset of jaundice (8.5 h [5.5, 22.5 h] vs. 38 h [34, 49.5 h], respectively) and had a higher incidence of hepatosplenomegaly (9% vs. 0%) (both *p* < 0.05). However, the incidences of hyperbilirubinemia (12% vs. 6%) and anemia (12% vs. 3%) were not significantly different between the two groups (both *p* > 0.05). Additionally, no cases of bilirubin encephalopathy occurred in either group.

#### 3.2.3. Laboratory Results

Compared with the non-RhD group, the RhD group exhibited significantly earlier peak times for RET (17 (5.75, 29.25) vs. 39 (23, 114)) and RDW-CV(23 (5.75, 36.5) vs. 48 (36, 120)) (both *p* < 0.05). Conversely, the peak TBIL value, time-to-peak TBIL value, HGB nadir value, peak RET value, and peak RDW-CV value were not significantly different among the two groups (all *p* > 0.05).

#### 3.2.4. Treatment Modalities and Outcomes

Compared with those in the non-RhD group, the length of hospital stay among participants in the RhD group was significantly longer (5 [4,5] days vs. 8.5 [6, 10.5] days, respectively; *p* < 0.05). There were no significant differences in the administration of IVIG, intensive phototherapy, albumin, blood transfusion, or exchange transfusion between the two groups (*p* > 0.05). Neither group had any cases of bilirubin encephalopathy or imaging abnormalities related to hyperbilirubinemia, and no significant difference was seen in the incidence of hearing abnormalities between the groups (*p* > 0.05).

### 3.3. Comparison Between the Single-Antibody and Multi-Antibody Subgroups

Among the 32 patients in the irregular antibody group, 24 had hemolysis caused by a single irregular antibody (single-antibody group), and 8 had multiple irregular antibodies (multi-antibody group). The multi-antibody group included one, one, two, two, one, and one patient with anti-C + anti-E, anti-C + anti-e, anti-E + anti-c, anti-C + anti-D, anti-C + anti-D + anti-E, and Kidd anti-Jka + Rh anti-E-induced hemolysis, respectively. Conversely, among the 20 patients in the RhD subgroup, 17 patients only had anti-D antibodies (anti-D single-antibody group), whereas 3 patients had anti-D combined with another non-anti-D antibody (anti-D multi-antibody group). Compared with the anti-D single-antibody group, the anti-D multi-antibody group exhibited earlier jaundice onset (*p* < 0.1). In addition, all patients in the anti-D multi-antibody group received intensive phototherapy, with 66.67% (2/3) requiring blood transfusion and exchange transfusion (*p* < 0.1). The multi-antibody group also showed higher peak values for RET and RDW-CV (*p* < 0.1), along with earlier time to occurrence (*p* < 0.05, Table 4).

### 3.4. Clinical Characteristics of Hemolytic Disease Caused by Rare Blood Type Incompatibility

In the irregular antibody group, in addition to 28 patients with hemolysis induced by Rh blood type incompatibility, one patient had a hemolytic disease caused by MNS blood type incompatibility, two patients had Kidd blood type incompatibility, and a patient had Kidd anti-Jka + Rh anti-E hemolytic disease. The characteristics of these four patients with hemolytic disease caused by rare blood type incompatibility are listed in Table 5.

### 3.5. HDN Prognosis

A total of 30/96 patients in the ABO incompatibility group and 21/32 patients in the irregular antibody group were followed up for between 1 week and 3 months after birth to monitor changes in HGB and outpatient treatment outcomes. The results revealed no significant between-group differences with respect to the incidence of late-onset anemia (41.38% vs. 57.14%) or mean hemoglobin levels between 1 week and 3 months after birth (108.56 ± 18.74 g/L vs. 103.52 ± 23.11 g/L) (both *p* > 0.05).

## 4. Discussion

### 4.1. Incidence of HDN Caused by Different RBC Antibodies

In the current study, 4.6% of HDN cases were caused by irregular antibodies. Hemolysis due to Rh anti-D antibodies was the most common cause, accounting for 62.5% of all cases. However, hemolysis caused by Rh non-anti-D antibodies accounted for 25%. The most common causative antibody was anti-D (62.5%), followed by anti-E (25%), anti-C (15.6%), anti-c (6.2%), and anti-e (3.1%). Hemolytic disease from other rare blood groups, such as MNS and Kidd, was only observed sporadically.

### 4.2. Fetal Status in HDN Caused by Irregular Antibodies

Our 13-year data revealed that most mothers of newborns in the irregular antibody group had a history of prior pregnancies. Almost all of these mothers tested positive for irregular antibodies during prenatal screening. However, only 16 in the irregular antibody group underwent MCA-PSV monitoring, with 3 showing an MCA-PSV value greater than 1.5 MoM. All of these mothers required preterm cesarean sections, and one mother (with anti-D antibody) required one session of intrauterine transfusion. Specifically, in the RhD group, only 10% of mothers received anti-D antibody injection therapy.

HDN primarily affects the fetus by causing varying degrees of anemia, which may lead to complications such as fetal hydrops and stillbirth. Pregnant women who screen positively for irregular antibodies should have their antibody titers regularly monitored. If the titers exceed the critical threshold, weekly MCA-PSV measurements should be taken to screen for fetal anemia. A previous study indicated that a value of 1.55 times the median MoM was the optimal threshold for detecting severe anemia, with a sensitivity of 100% and a false-positive rate of 12% [5]. At this point, other indicators of fetal anemia should be considered to determine if fetal blood sampling is required to assess fetal hemoglobin levels and decide whether intrauterine transfusion is necessary [3]. In the present study, only half of the pregnant women with positive antibody test results underwent MCA-PSV monitoring, highlighting the insufficient awareness and inadequate screening for HDN.

### 4.3. Clinical Characteristics of HDN Caused by Irregular Antibodies

The results of the present study revealed that newborns with irregular antibody-induced HDN exhibited an earlier onset of jaundice, had a higher rate of receiving intensive phototherapy and exchange transfusions, and experienced longer hospital stays. Zheng et al. [6] further compared the average daily bilirubin levels in 67 cases of non-ABO HDN and 200 cases of ABO-HDN. The results revealed that bilirubin levels in infants with non-ABO HDN increased more quickly, peaked higher, and persisted longer. After treatment using blue light phototherapy and IVIG administration to block antibodies and inhibit hemolysis, the symptoms were alleviated in 59 cases. Additionally, eight cases required exchange transfusions due to high bilirubin levels, and we found consistent data. In the current study, the irregular antibody group developed jaundice earlier. Furthermore, the risk of complications associated with neonatal hyperbilirubinemia was correlated with the time of jaundice onset. The earlier the incidence of neonatal hyperbilirubinemia, the higher the risk of developing complications for the infant.

The administration of IVIG treatment was not significantly different between the ABO incompatibility and irregular antibody groups. The timing and indications for IVIG in HDN remain controversial [7]. One study has suggested that IVIG may increase the risk of necrotizing enterocolitis in full-term infants [8]. A 2018 Cochrane meta-analysis by Zwiers et al. [9] assessed the evidence for this recommendation and concluded that there was insufficient evidence to show that IVIG could prevent the need for exchange transfusion. As such, they recommended that IVIG should only be considered in facilities where exchange transfusions were not possible. In our center, for infants diagnosed with HDN caused by ABO incompatibility, IVIG treatment is considered only when serum bilirubin levels significantly increase to the threshold for exchange transfusion, and either phototherapy is ineffective or progressive anemia suggests a need for transfusion.

The present study found that the irregular antibody group exhibited not only earlier onset of anemia but also greater severity compared with the other groups. Additionally, the group had a higher incidence of hepatosplenomegaly and required more frequent blood transfusions. One patient with HDN caused by anti-M antibodies presented with hypovolemic shock immediately after birth. HDN can lead to early-onset anemia (within the first week of life) or late-onset anemia. Late-onset anemia, which typically affects infants with HDN born at or after 35 weeks, can be further categorized into advanced hemolytic anemia and advanced aplastic anemia, depending on the underlying mechanism [10]. Maternal alloantibodies can persist in the infant’s circulation for up to 6 months postpartum, continually triggering antigen–antibody reactions that cause hemolytic anemia. Certain antibodies, such as anti-Kell alloantibodies and anti-M antibodies, can inhibit erythropoiesis in addition to inducing antigen–antibody reactions, potentially leading to aplastic anemia lasting up to 3 months [11]. Moreover, several studies [12,13] have discovered a link between the presence of RBC antibodies in breast milk and prolonged neonatal hemolysis and anemia. Wang et al. [14] also reported a case of severe neonatal anemia caused by anti-C antibodies, where prenatal signs were limited to polyhydramnios. Postpartum, the infant required multiple blood transfusions due to severe anemia. Other studies [15,16] noted that anti-M antibodies could result in aplastic anemia, with reduced RET counts indicating insufficient hematopoietic activity in these cases. Rh incompatibility hemolytic disease can also result in aplastic anemia [17]. The current study analyzed the follow-up data from birth up to 3 months of 51 infants. The results revealed no significant difference between the irregular antibody group and the ABO incompatibility group with respect to the incidence or severity of late-onset anemia. Owing to the limited sample size, larger and longer-term follow-up studies are required for further validation. In cases of severe, unexplained anemia after birth, screening for irregular antibodies should be prioritized. Upon the confirmation of antibody-mediated hemolytic anemia, the patient should be closely monitored for hemoglobin fluctuations in the first 6 months of life, with transfusions administered as necessary to prevent organ damage caused by late-onset anemia.

Regarding laboratory findings, we found that RET and RDW-CV values peaked earlier in the irregular antibody group than in the ABO incompatibility group and that these peaks occurred significantly earlier than the appearance of HGB nadir. During hemolysis, massive RBC destruction leads to reduced oxygen tension in the bloodstream, prompting immature RBCs to enter the circulation prematurely, which results in elevated RET levels. Therefore, RETs are an effective marker for HDN diagnosis [18]. Elfarargy et al. [19] reported that the RET percentage in umbilical cord blood (with a critical value of 5.7%) could be used as an early predictor of neonatal hyperbilirubinemia. Similarly, Yang et al. [20] found that RET percentage was a sensitive indicator for monitoring the progress of treatment for HDN, with a significant decrease in RETs indicating treatment efficacy. RDW is a parameter reflecting the size heterogeneity of peripheral RBCs, with an increased RDW being associated with more extensive RBC destruction and ineffective erythropoiesis [21]. Our results indicate that if the RETs and RDW-CV in neonates with hemolytic disease increase rapidly within 24–48 h of birth, the possibility of hemolysis due to irregular antibodies should be considered even in the absence of a significant decrease in HGB. Additionally, among the 32 patients with hemolysis caused by irregular antibodies, 31 patients exhibited DAT positivity, with only 1 patient with MNS blood group incompatibility showing a negative DAT. In their analysis of 67 cases of non-ABO HDN, Zheng et al. [6] found that, except for two cases involving anti-M antibodies, one case of anti-Jka antibodies, and two cases of anti-E antibodies combined with ABO HDN showing weak DAT positivity, all other cases had strongly positive DAT results (≥2+). Consistent findings were observed in the current study. Given that IAT is yet to be available in many primary hospitals in China, a negative DAT result may serve as an approximate indicator to rule out irregular antibodies, although the possibility of hemolysis caused by anti-M antibodies should be considered.

### 4.4. Comparison of Hemolysis Between Single- and Multi-Antibody Groups

In the irregular antibody group, eight patients had hemolysis resulting from the simultaneous presence of multiple irregular antibodies, in addition to 24 patients with hemolysis caused by a single irregular antibody. These findings suggest that the presence of multiple antibodies in the Rh blood group system leads to more severe clinical manifestations compared with the presence of a single antibody.

Zeynep et al. [22] found a higher incidence of HDN in pregnancies with anti-D antibodies combined with another RBC alloantibody than in those with isolated anti-D immunization. Furthermore, the concurrent presence of anti-D and non-D Rh antibodies led to greater HDN severity, necessitating more invasive treatment procedures. Singh et al. [23] investigated 176 pregnant women with positive anti-D antibodies and discovered that 77.3% had only anti-D antibodies, whereas 22.7% had at least one additional antibody. Similarly, they reported that patients with additional antibodies had a 1.7-fold higher relative risk of severe HDN, although the exact mechanism remained unclear. However, they speculated that this increased risk may be associated with additional antibodies binding to fetal and neonatal RBCs, leading to heightened secondary hemolysis. Nevertheless, not all combinations of RBC antibodies will result in aggravated hemolysis. Two studies [24,25] suggested that ABO incompatibility may offer a protective effect against Rh sensitization. Rh antibody production is characterized by a sufficiently long survival time of RBCs in the maternal circulation, which stimulates the production of anti-D antibodies. If fetal RBCs are incompatible with maternal anti-A or anti-B antibodies, they will be rapidly hemolyzed, preventing the Rh antigen from generating an antigenic stimulus. The impact of multiple RBC antibodies on hemolysis may depend on the types of antibodies present. Further investigation is warranted regarding the underlying mechanisms of this phenomenon.

### 4.5. Significance of Early Screening and Intervention for Irregular Antibodies

Our results indicate that although the incidence of HDN caused by irregular RBC antibodies is rare, the condition nevertheless poses significant risks to neonates. Moreover, HDN is more prevalent among pregnant women with a history of previous pregnancies, miscarriages, or past occurrences of HDN. Considering the national fertility policy reforms in China, the number of HDN cases is expected to increase continuously, making it crucial to promptly identify at-risk pregnancies for effective management of HDN. Currently, pregnant women in China are required to undergo ABO and RhD blood typing and screening for irregular antibodies during their initial visit at 8 weeks to 12 weeks of gestation. If antibody screening yields positive results, the specific antibodies must be determined, and antibody titers should be assessed. If the results are negative, a retest is conducted at 28 weeks of gestation [26]. For pregnant women with clinically significant irregular antibodies, performing umbilical blood tests on neonates after delivery to determine blood type, HGB levels, RET, and DAT as early as possible is essential, in addition to strengthening intrauterine monitoring. This approach may allow the timely detection and treatment of HDN, thereby reducing the risk of severe complications.

This study has several limitations. First, establishing causality in certain cases of positive DAT is challenging owing to the retrospective nature of the study. The weak positive DAT results observed in some patients may be false positives, limiting the availability of sufficient clinical data for analysis. Second, this study was considerably long, and diagnostic and intervention standards for neonatal hyperbilirubinemia may have evolved during the study period. Moreover, for controversial treatment methods such as IVIG, different physicians at the authors’ center may have applied varying standards. Finally, the sample size is small, and the patients were included from a single center. Larger, multi-center prospective studies are needed for further verification.

### 4.6. Other Factors That May Affect the Severity of HDN and Future Research Directions

Antigenic density can vary significantly across different blood group systems. High antigen density increases immunogenicity and increases the potential for maternal antibody production, leading to HDN [27]. In contrast, Rh antigens are expressed at a much lower density; however, despite this, the immunogenicity of Rh antigens, particularly RhD, remains significant. This discrepancy in antigen density between different blood group systems may contribute to the clinical severity and timing of HDN, with ABO incompatibility typically resulting in less severe disease during the first pregnancy, while Rh incompatibility is associated with more severe disease in subsequent pregnancies [28]. The ability of different IgG subclasses to pass through the placenta and bind to Fc receptors on macrophage membranes varies, as does their hemolytic activity. IgG2, commonly found in the maternal serum, has relatively weak hemolytic activity, whereas IgG1 and IgG3 have stronger hemolytic activity in addition to the ability to cross the placenta, meaning that even at lower concentrations, they can cause significant hemolysis [29]. Brouwers et al. [30] previously found that the antibody-dependent cell-mediated cytotoxicity (ADCC) assay using maternal serum was the most sensitive assay to predict ABO-HDN, while the combination of the ADCC assay with A or B antigen density determination was the most specific test. The possibility of predicting the severity of HDN through the early monitoring of red blood cell antigen density in maternal or umbilical cord blood, as well as the subtypes of antibodies, is an area of ongoing research.

## 5. Conclusions

HDN caused by irregular red blood cell antibodies is rare, but clinical manifestations are serious. It is important to pay attention to the screening of irregular antibodies during pregnancy, strengthen monitoring, and provide intrauterine treatment and early intervention as necessary. Anemia caused by HDN can affect the first 3 months of life, and the follow-up of these patients should be strengthened.

## Figures and Tables

**Table 1:** Comparison of clinical manifestations between the ABO incompatibility and irregular antibody groups.

Group/Size	ABO Incompatibility Group (n = 96)	Irregular Antibody Group (n = 32)	χ^2^ or U-Value	*p*-Value
Hepatosplenomegaly, n (%)	5 (5.21)	9 (28.12)	10.693	**0.001**
Hyperbilirubinemia, n (%)	66 (68.75)	21 (65.62)	0.108	0.743
Anemia, n (%)	35 (36.46)	18 (56.25)	3.875	**0.049**
Time to onset of jaundice (h), median (range)	25 (20, 36)	19.5 (7.75, 36)	2.098	**0.036**

**Table 2:** Comparison of laboratory results between the ABO incompatibility and irregular antibody groups.

Group/Size	ABO Incompatibility Group (n = 96)	Irregular Antibody Group (n = 32)	χ^2^, t, or U-Value	*p*-Value
DAT positivity, n (%)	60 (62.50)	31 (96.88)	12.80	**<0.001**
Peak TBIL value (mg/dL), median (range)	14.70 (12.78, 17.80)	14.80 (12, 16.92)	−0.105	0.917
Time-to-peak TBIL value (d), median (range)	3 (2, 4)	4 (2, 5)	−0.997	0.319
HGB nadir value (g/L), median (range)	136.59 ± 25.69	115.38 ± 36.61	3.038	**0.004**
Time to HGB nadir (h), median (range)	87 (48, 156)	48 (20, 143.75)	2.045	**0.041**
Peak RET value (%), median (range)	6.14 (4.38, 7.46)	6 (3.47, 9.53)	−0.025	0.98
Time-to-peak RET value (h), median (range)	36 (26, 60)	24 (10, 42)	2.716	**0.007**
Peak RDW-CV value (%)	16.90 (16, 18.3)	16.25 (15.57, 17.55)	1.245	0.213
Time-to-peak RDW-CV value (h), median (range)	38 (27.5, 72)	25.5 (13.5, 48)	2.427	**0.015**

DAT, direct antiglobulin test; TBIL, total bilirubin; HGB, hemoglobin; RET, reticulocyte; RDW-CV, coefficient of variation of red blood cell distribution of width.

**Table 3:** Comparison of treatment modalities and outcomes between the ABO incompatibility and irregular antibody groups.

Group/Size	ABO Incompatibility Group (n = 96)	Irregular Antibody Group (n = 32)	χ^2^, t, or U-Value	*p*-Value
IVIG, n (%)	47 (48.96)	22 (68.75)	3.784	0.052
Intensive phototherapy, n (%)	16 (16.67)	14 (43.75)	9.81	**0.002**
Albumin infusion, n (%)	17 (17.71)	7 (21.88)	0.274	0.601
Blood transfusion, n (%)	1 (1.04)	8 (25)	17.569	**<0.001**
Exchange transfusion, n (%)	3 (3.12)	6 (18.75)	6.733	**0.009**
Length of hospital stay (d), median (range)	5.5 (4, 8)	6.5 (5, 10)	−2.03	**0.042**
Hearing abnormalities, n (%)	2 (2.08)	1 (3.12)	*	1
Imaging anomalies, n (%)	4 (4.17)	1 (3.12)	0	1.000

* Fisher’s exact test. IVIG, intravenous immunoglobulin.

**Table 4:** Comparison of laboratory results between the anti-D single-antibody and anti-D multi-antibody subgroups.

Group/Size	Anti-D Single-Antibody Subgroup (n = 17)	Anti-D Multi-Antibody Subgroup (n = 3)	U- or t-Value	*p*-Value
Time to jaundice onset (h), median (range)	10 (7, 24)	3 (2.5, 5.5)	1.859	0.063
Peak RET value (%), median (range)	5.77 (3.49, 9.78)	13.63 (11.32, 23.38)	−1.747	0.081
Time-to-peak RET value (h), median (range)	25.65 ± 19.01	5.67 ± 3.06	4.047	**0.001**
Peak RDW-CV value (%)	16.3 (15.2, 18.2)	22.4 (19.45, 24.6)	−1.907	0.056
Exchange transfusion, n (%)	2 (11.76%)	2 (66.67%)	*	0.088

* Fisher’s exact test. RET, reticulocytes; RDW-CV, coefficient of variation of red blood cell distribution of width.

**Table 5:** Characteristics of the four patients with hemolytic disease caused by rare blood type incompatibility.

No.	Maternal Gravidity/Parity (n)	Sex	Blood Type	Maternal Blood Type	DAT/ IAT	Antibody Type	Time to Jaundice Onset	Peak TBIL (mg/dL)/Time to Peak	Nadir HGB (g/L) /Time to Nadir Age	Peak RET (%) /Time to Peak	Length of Hospital Stay (d)	Treatment Modality
1	2/1	Male	B, Rh (D)+ MN	O, Rh (D)+ NN	**Negative**/ positive	Anti-M	19 h	15.4/40 h	57/at birth	0.67/4 d	6	Standard phototherapy, transfusion
2	2/2	Male	B, Rh (D)+ Jk (a^+^b^+^)	B, Rh (D)+ Jk (a^+^b^−^)	Positive/ positive	Anti-Jkb	12 h	16.6/24 h	103/24 h	6.76/24 h	5	Intensive phototherapy, IVIG, albumin, exchange transfusion
3	3/2	Male	B, Rh (D)+ Jk (a^+^b^+^)	B, Rh (D)+ Jk (a^−^b^+^)	Positive/ positive	Anti-Jka	36 h	14.2/5 d	101/53 d	6.63/60 h	5	Standard phototherapy
4	7/4	Male	A, Rh (ccDEe) Jk (a^+^b^+^)	AB, Rh (ccDee) Jk (a^−^b^+^)	Positive/ positive	Anti-E, Anti-Jka	31 h	11/32 h	148/40 h	6.23/40 h	5	Standard phototherapy

IVIG, intravenous immunoglobulin; RET, reticulocytes; HGB, hemoglobin; DAT, direct antiglobulin test; IAT, indirect antiglobulin test.

## Data Availability

The data presented in this study are only available on request from the corresponding author due to the author’s workplace policy on medical data.
